# Arrest at the diplotene stage of meiotic prophase I is delayed by progesterone but is not required for primordial follicle formation in mice

**DOI:** 10.1186/s12958-016-0218-1

**Published:** 2016-12-05

**Authors:** Sudipta Dutta, Deion M. Burks, Melissa E. Pepling

**Affiliations:** 1 Department of Biology, Syracuse University, 107 College Place, Syracuse, NY 13244 USA; 2 Present Address: University of Michigan Ann Arbor, Ann Arbor, MI USA

**Keywords:** Fetal oocyte development, Steroid hormones, Diplotene arrest, Meiotic prophase I progression, Primordial follicle formation

## Abstract

**Background:**

In mammalian females, reproductive capacity is determined by the size of the primordial follicle pool. During embryogenesis, oogonia divide mitotically but cytokinesis is incomplete so oogonia remain connected in germ cell cysts. Oogonia begin to enter meiosis at 13.5 days postcoitum in the mouse and over several days, oocytes progress through the stages of meiotic prophase I arresting in the diplotene stage. Concurrently, germ cell cysts break apart and individual oocytes become surrounded by granulosa cells forming primordial follicles. In rats, inhibition of a synaptonemal complex protein caused premature arrival at the diplotene stage and premature primordial follicle assembly suggesting diplotene arrest might trigger primordial follicle formation. Cyst breakdown and primordial follicle formation are blocked by exposure to steroid hormones but hormone effects on the timing of diplotene arrest are unclear. Here, we asked: (1) if oocytes were required to arrest in diplotene before follicles formed, (2) if all oocytes within a germ cell cyst arrested at diplotene synchronously, and (3) if steroid hormones affected progression through prophase I.

**Methods:**

Meiotic stage and follicle formation were assessed in histological sections. Statistical differences over time were determined using one-way ANOVA followed by Newman-Keuls multiple comparisons test. To determine if steroid hormones affect the rate of progression to the diplotene stage, 17.5 dpc ovaries were placed in organ culture with media containing estradiol, progesterone or both hormones. In this case, differences were determined using one-way ANOVA followed by Dunnett’s multiple comparisons test.

**Results:**

We found primordial follicles containing oocytes at the diplotene stage as well as follicles containing oocytes at pre-diplotene stages. We also found individual germ cell cysts containing oocytes at both diplotene and pre-diplotene stages. Progesterone but not estradiol reduced the number of diplotene oocytes in ovary organ culture.

**Conclusions:**

Our results suggest that meiotic progression and primordial follicle formation are independent events. In addition, oocytes in germ cell cysts do not synchronously proceed through meiosis. Finally, only progesterone delayed transit though meiotic prophase I.

## Background

In female mammals, reproductive capacity is determined at birth by the non-renewable pool of primordial follicles present, representing the total population of germ cells available for reproductive purposes [1]. In mice, the primordial germ cells migrate to the genital ridge and divide by mitosis until 13.5 days postcoitum (dpc) [2]. During these divisions the germ cells are known as oogonia and develop in germ cell cysts due to incomplete cytokinesis following each cell cycle [3]. Oogonia start to enter meiosis at approximately 13.5 dpc and are then referred to as oocytes [2]. Oogonia do not appear to enter meiosis synchronously [2, 4]. However, meiosis proceeds from anterior to posterior suggesting that local factors diffuse from the mesonephros at the anterior side of the ovary to promote meiosis [5]. Most germ cells have entered meiosis by 15.5 dpc [2]. After entering meiosis oocytes progress through the initial stages of meiotic prophase I and remain arrested at the diplotene stage until just prior to ovulation. Some oocytes arrive at the diplotene stage by 17.5 dpc but it takes several days until all oocytes are in diplotene [2]. During the same time period, germ cell cysts break apart and individual oocytes become surrounded by granulosa cells forming primordial follicles [6]. Some follicles, referred to as the first wave of developing follicles are activated to grow immediately after forming while most follicles are not activated until sexual maturity developing in groups in a cyclical fashion [7, 8].

Mutations in genes responsible for the initial stages of meiosis in mouse such as disrupted meiotic cDNA 1 (*Dmc1*) and meiosis-specific sporulation protein (*Spo11*) result in loss of oocytes and an inability to form follicles which ultimately cause sterility [9, 10]. Meiotic prophase I is marked by the expression of synaptonemal complex proteins (SYCPs) that make up the synaptonemal complex which is required for DNA synapsis and meiotic recombination between homologous chromosomes. In rats, inhibition of SYCP1 accelerated arrival at the diplotene stage along with premature assembly of those oocytes into primordial follicles [11]. In fetal bovine ovaries, many oocytes do not appear to arrest at diplotene but instead continue on through diakinesis where they are eventually lost by attrition [12]. A crucial factor for the survival of germ cells may be their ability to be enclosed within follicles [13]. This evidence reflects the possibility of a link between two events that occur during early mouse oogenesis, arrest at the diplotene stage and primordial follicle formation.

Both estradiol (E_2_) and progesterone (P_4_) can delay cyst breakdown and primordial follicle formation [14]. Estrogens have also been shown to affect meiotic progression of oocytes. When pregnant mice were treated with bisphenol A (BPA), a known estrogenic compound, oocytes from female fetuses of exposed mothers had synaptic defects and recombination aberrations [15]. In adults, those aberrations gave rise to aneuploid eggs and embryos. In cattle, it is thought that primordial follicles cannot be activated until after the oocyte reaches diplotene arrest. In vitro treatment of bovine ovaries with estradiol (E_2_) or progesterone (P_4_) decreased the number of follicles that were activated and thus, may affect progression to the diplotene stage [16].

The main objectives of the present study were to determine if diplotene arrest is linked with primordial follicle formation in mice and to elucidate the role of steroid hormone signaling in meiotic progression of murine oocytes. The molecular mechanisms involved in regulating progression through prophase I and in primordial follicle formation in the developing ovary are still poorly understood. Elucidating events during fetal ovarian development will increase our understanding of the factors that control oocyte quality and quantity and thus help in improvement of current infertility treatments.

## Methods

### Animals

Adult CD1 male and female outbred mice were obtained from Charles River Laboratories (Wilmington, MA, USA) and were maintained in accordance with the policies of Syracuse University’s Institutional Animal Care and Use Committee. Mice were housed and bred at a controlled photoperiod (14 h light, 10 h dark), temperature (21–22 °C), and humidity with food and water available ad libitum. CD1 females were mated with males of the same strain and checked daily for vaginal plugs. Noon on the day of vaginal plug detection was designated as 0.5 dpc. Birth usually occurred at 19.5 dpc and was designated as postnatal day (PND) 1. Pregnant mice were euthanized by CO_2_ asphyxiation for fetal ovary collection. For neonatal ovary collection, pups were euthanized by decapitation on the appropriate day.

### Histological methods

Fetal and neonatal ovaries were dissected and trimmed of extra tissue. They were fixed in Bouin’s solution for 2 h at room temperature, followed by dehydration through an ethanol series. Histological processing of the ovaries was performed at the Electron Microscopy & Histology Core Facility, Weill Cornell Medical College, New York. Following standard protocols for paraffin-embedded sections, ovaries were serially sectioned at 5 μm and stained with hematoxylin and eosin. Images were taken on an Olympus BX50 microscope with an Olympus DP71 digital camera. Every fifth section was marked for direct counts of oocytes at pre-diplotene and diplotene stages and four to five ovaries were analyzed at each age. A total of 10–12 sections per ovary were used for counting. To avoid bias, all ovaries were analyzed without knowledge of age. To avoid double counting of oocytes, only oocytes having a visible nucleus were counted. Oocytes at pre-diplotene stages were characterized at the leptotene stage by the appearance of thin long threads of chromatin, at the zygotene stage by regions of thicker chromosome strands and at the pachytene stage by thick highly condensed chromatin. Oocytes at the diplotene stage were recognized by areas of condensed chromatin interspersed with clear areas [17]. The total number of pre-diplotene and diplotene oocytes for each ovary was determined by multiplying the number of pre-diplotene and diplotene oocytes by five to account for every fifth section being used in the analysis. Oocytes were counted as cysts if they were present in clusters of at least two oocytes without any intervening somatic cells. Oocytes were considered to be in primordial follicles if they contained an oocyte surrounded by a layer of flattened granulosa cells.

### In vitro ovary organ culture

Ovaries dissected at 17.5 dpc were placed in culture. Ovaries were cultured in 4-well culture plates in drops of media on 0.4 μM floating filters (Millicell- CM; Millipore Corp., Bedford, MA) in 0.4 ml DMEM-Ham’s F-12 media supplemented with penicillin-streptomycin, 5X ITS-X (Life Technologies, Inc., Grand Island, NY), 0.1% BSA, 0.1% albumax, and 0.05 mg/ml L-ascorbic acid. E_2_ and P_4_ (Sigma Chemical Co., St. Louis, MO) were dissolved in dimethylsulfoxide (DMSO) at a concentration of 0.1 M and then added to culture media to achieve the desired final concentration. DMSO was added to media at the same percent as the chemical to serve as vehicle control. Ovaries were placed in culture and exposed daily to E_2_, P_4_ or both hormones at 10^−6^ M or DMSO alone (*n* = 5 ovaries per treatment group). Ovaries were divided randomly among the treatment groups. The ovaries from control and treatment were fixed in Bouin’s fixative and histologically processed.

### Statistical analysis

The percent of oocytes in diplotene, percent of oocytes in follicles and percent of follicles containing pre-diplotene oocytes over time were calculated using four to five ovaries at each time point. Data are represented as mean ± SEM of nontransformed data. As counted data by nature are non-normally distributed, logarithmic transformation (Y = log [y]) was performed on the data. Statistical analyses using transformed data were performed using GraphPad Prism version 6 (GraphPad Software, San Diego, CA). Statistical differences (*P* < 0.05) among the means were evaluated using one-way ANOVA followed by Newman-Keuls multiple comparisons test. Effects of E_2_ and P_4_ on oocyte number, percent single oocytes, percent oocytes in diplotene and percent of follicles containing diplotene oocytes were analyzed using one-way ANOVA followed by Dunnett’s multiple comparisons test using five ovaries per treatment group. All results are presented as mean ± SEM of nontransformed data.

## Results

### Meiotic progression and diplotene arrest in developing mouse ovaries

**Fig. 1:**
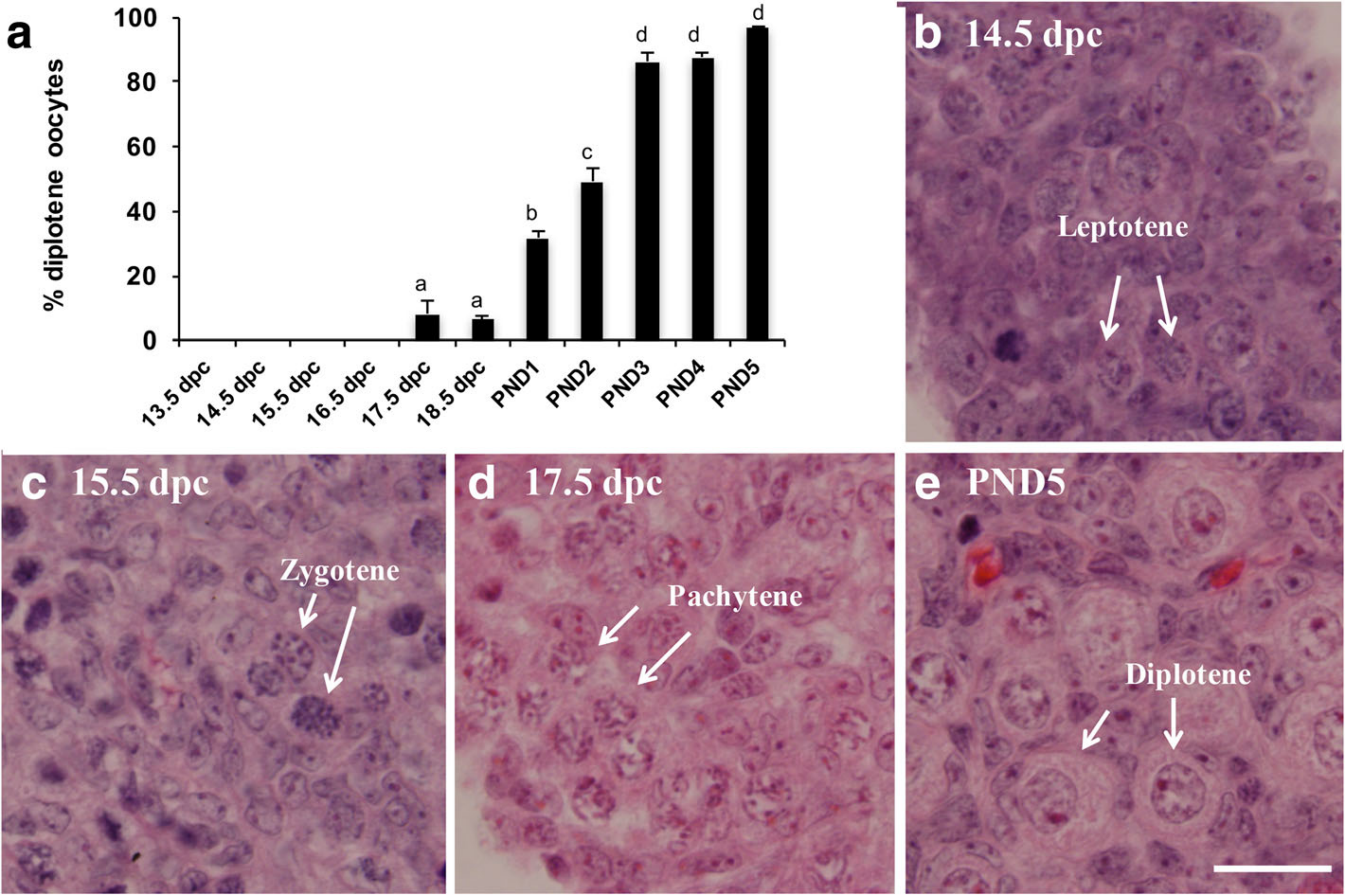
Diplotene stage oocytes are first detected at 17.5 dpc. **a** Percentage of oocytes at the diplotene stage of meiotic prophase I (± SEM) in perinatal mouse ovaries from 13.5 dpc to PND5. Different letters indicate a significant difference between groups (*P* < 0.05; *n* = 4–5 ovaries per time point) as determined by one-way ANOVA followed by Newman-Keuls multiple comparisons test. **b-e** Representative histological sections of perinatal mouse ovaries stained with hematoxylin and eosin showing different meiotic prophase I stages indicated by white arrows. **b** 14.5 dpc ovary showing oocytes at leptotene. **c** 15.5 dpc ovary showing oocytes at zygotene. **d** 17.5 dpc ovary showing oocytes at pachytene. **e** PND5 ovary showing oocytes at diplotene. Scale bar = 20 μM

To investigate the relationship between meiotic progression and primordial follicle formation, we first performed a quantitative study on histological sections from 13.5 dpc to PND5, determining the number of oocytes at each meiotic stage. The first diplotene oocytes were observed at 17.5 dpc, when about 8% of the oocytes were at the diplotene stage (Fig. 1a). A statistically significant increase to 32% diplotene stage oocytes was observed at PND1, to 49% at PND2 and to 86% at PND3. A slight but non-significant increase was observed in PND5 ovaries.

Representative histological sections with examples of the different meiotic prophase I stages are shown in Fig. 1b–1e. Figure 1b shows a section from a 14.5 dpc ovary with oocytes at the leptotene stage identified by long thin nuclear threads as the chromatin begins to condense [16, 17]. Figure 1c shows a section from a 15.5 dpc ovary with oocytes at the zygotene stage characterized by regions of thicker chromatin as homologous chromosomes begin to pair. Figure 1d shows a 17.5 dpc ovary with oocytes at the pachytene stage distinguished by very thick strands of chromatin. Figure 1e shows a PND5 ovary with oocytes at diplotene characterized by areas of condensed chromatin separated by clear areas [17].

### Primordial follicle formation and diplotene arrest are independent processes

**Fig. 2:**
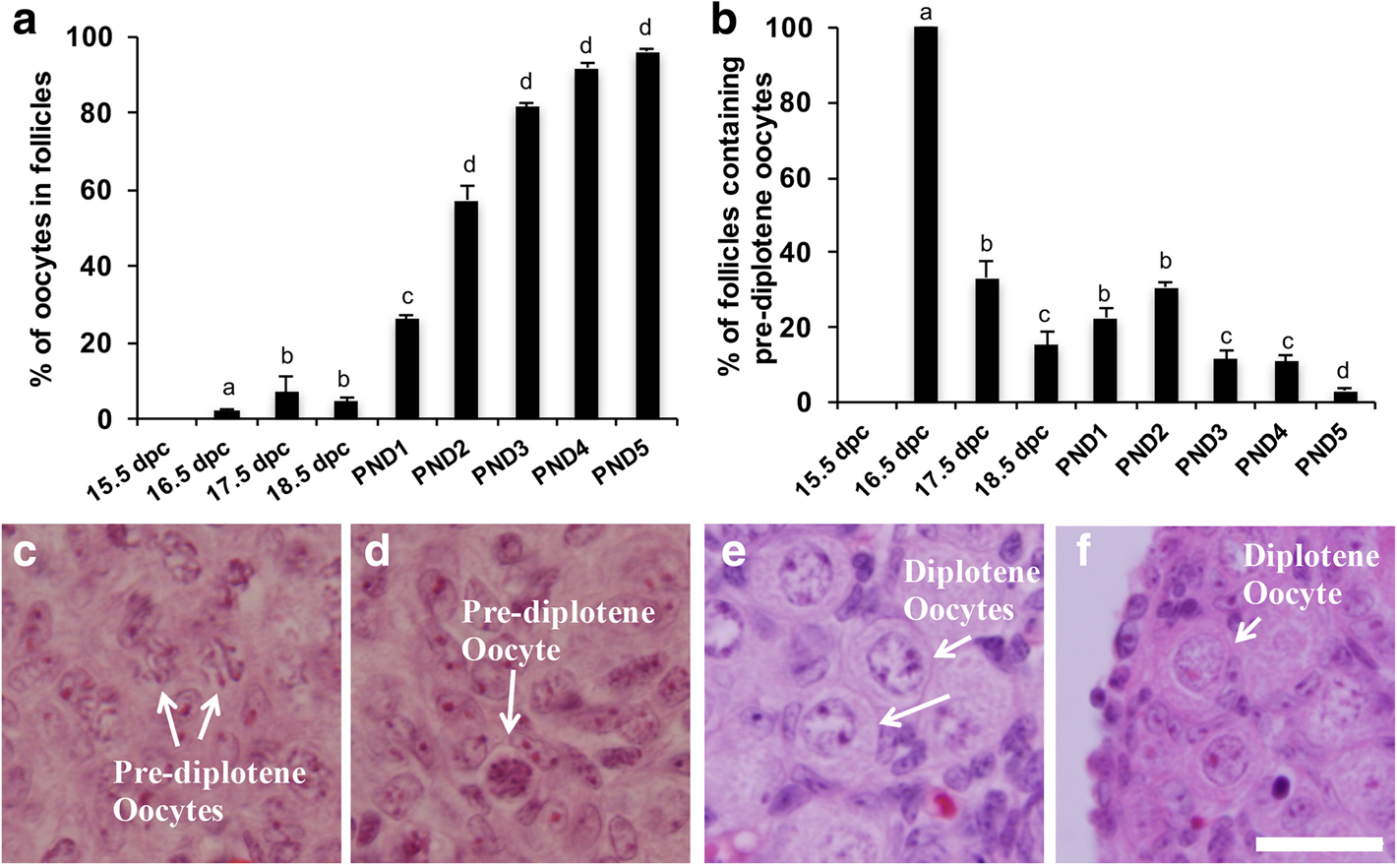
Primordial follicle formation and meiotic progression in perinatal mouse ovaries. **a** Percentage of oocytes in follicles (± SEM) over time. Different letters indicate a significant difference between groups (*P* < 0.05; *n* = 4–5 ovaries per time point) as determined by one-way ANOVA followed by Newman-Keuls multiple comparisons test. **b** Percentage of follicles with oocytes at the pre-diplotene stage out of total follicles (± SEM) in perinatal mouse ovaries. Different letters indicate a significant difference between groups (*P* < 0.05; *n* = 4–5 ovaries per time point) as determined by one-way ANOVA followed by Newman-Keuls multiple comparisons test. **c-f** Representative histological images of perinatal mouse ovaries stained with hematoxylin and eosin. **c** 17.5 dpc ovary showing pre-diplotene oocytes in germ cell cysts indicated by white arrows. **d** 17.5 dpc ovary showing a primordial follicle containing a pre-diplotene oocyte indicated by a white arrow. **e** PND3 ovary showing diplotene oocytes not yet enclosed in primordial follicles indicated by white arrows. **f** PND5 ovary showing a diplotene oocyte in a primordial follicle indicate by a white arrow. Scale bar = 20 μM

Previous studies from our lab have indicated that in the medullary region, primordial follicles are found as early as 17.5 dpc [6]. Here we performed a thorough investigation analyzing serial sections 5 μm apart covering the entire ovary thereby enabling us to obtain a more accurate estimate regarding the presence of primordial follicles. The earliest primordial follicles were detected at 16.5 dpc where we found 2% of the oocytes enclosed in follicles (Fig. 2a). We hypothesized that oocytes needed to reach diplotene before becoming enclosed in follicles. However, we observed pre-diplotene oocytes both in germ cells cysts (Fig. 2c) and in primordial follicles (Fig. 2d). Figure 2b shows the percentage of follicles containing pre-diplotene oocytes from 15.5 dpc to PND5. All of the follicles observed at 16.5 dpc were at the pre-diplotene stage. For all later stages, only a small percentage of follicles contained pre-diplotene oocytes with the majority already at diplotene (Fig. 2f). We also observed oocytes still in germ cell cysts that were at the diplotene stage (Fig. 2e). Thus, meiotic progression and primordial follicle formation do not appear to be linked.

### All oocytes in a cyst do not reach the diplotene stage synchronously

**Fig. 3:**
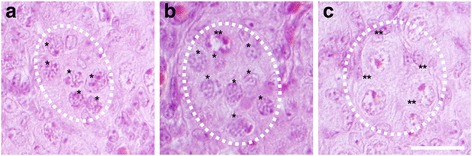
Representative images showing paraffin sections of PND1 mouse ovaries stained with hematoxylin and eosin. **a** Ovarian section showing a cyst with all pre-diplotene oocytes circled with a white dashed line. **b** Ovarian section showing a cyst with both pre-diplotene and diplotene stage oocytes circled by a white dashed line. **c** Ovarian section showing a cyst with all diplotene oocytes circled with a white dashed line. Pre-diplotene oocytes are indicated by one asterisk and diplotene oocytes are indicated by two asterisks. Scale bar = 20 μM

Oocytes enter meiosis in a wave from anterior to posterior in the mouse ovary. However, it is not known within individual cysts whether oocytes enter or progress through meiosis and arrest at diplotene synchronously. To determine if all oocytes in a germ cell cyst were at the same meiotic stage, a quantitative analysis of meiotic stages of oocytes present within individual cysts was performed at PND1. We chose this age because many pachytene and diplotene stage oocytes are present and most oocytes are still in germ cell cysts. For each cyst we determined whether an oocyte was at the pre-diplotene or diplotene stage of meiosis. We examined sections from five different ovaries and found cysts with only diplotene oocytes, with only pre-diplotene oocytes and with a mixture of pre-diplotene and diplotene oocytes (Fig. 3). For each cyst that had a mixture of different stage oocytes we determined the percent of oocytes at each stage. Each cyst contained two to five oocytes visible in the section. We found that individual cysts had an average of 63% diplotene oocytes. It is important to note that only oocytes in individual sections were analyzed and that the cysts likely contained more oocytes not visible in the section. These findings suggest that oocytes within individual cysts do not progress through meiosis synchronously.

### Effects of steroid hormones on meiotic progression

**Fig. 4:**
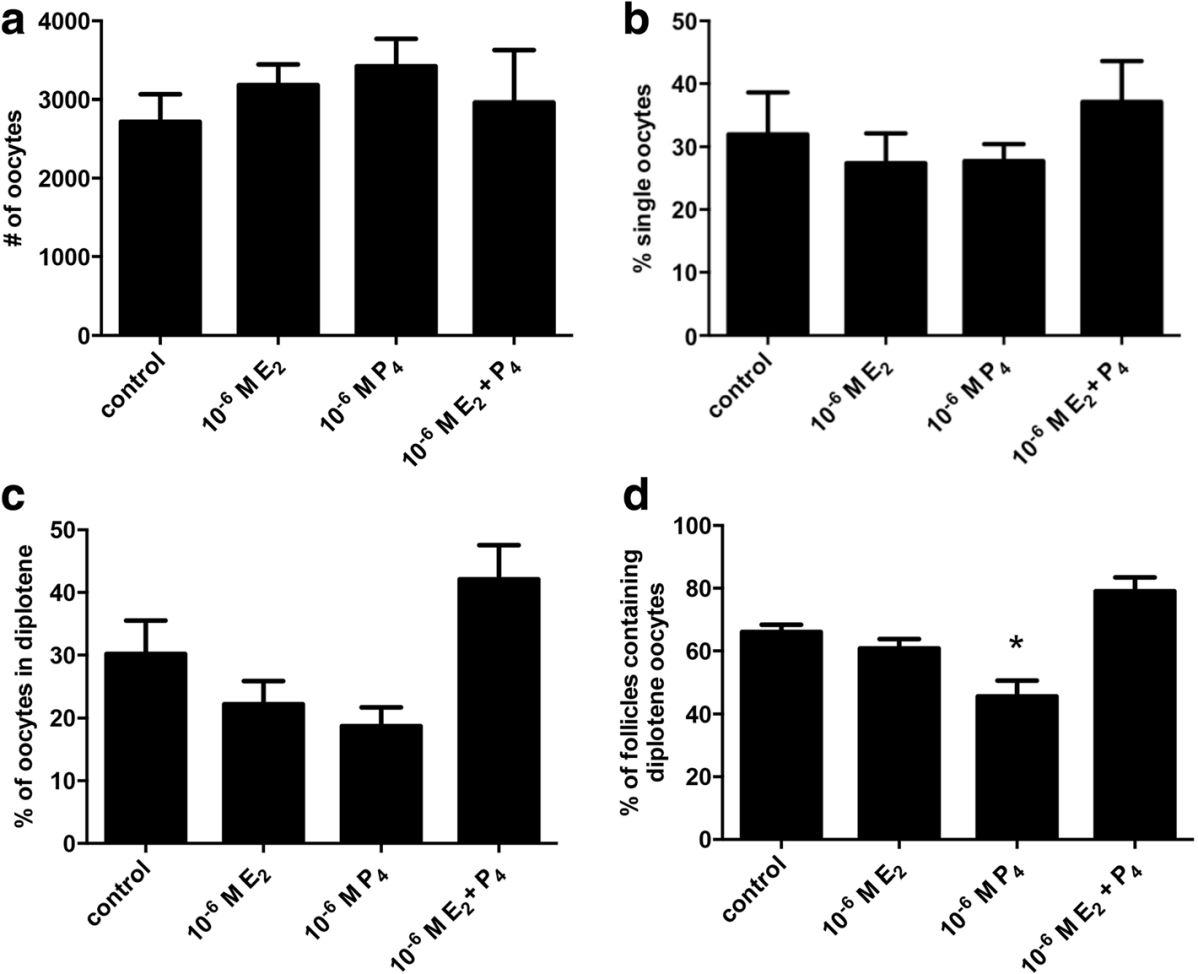
Effects of E_2_ and P_4_ treatment in organ culture on meiotic stage. **a** Total number of oocytes, **b** percent single oocytes **c** percent diplotene oocytes and **d** percent follicles containing diplotene stage oocytes per ovary in control ovaries or ovaries treated with 10^−6^ M E_2_, 10^−6^ M P_4_ or 10^−6^ M E_2_ + 10^−6^ M P_4_ for 4 days in organ culture. Data are presented as the mean ± SEM. * indicates a significant difference from control ovaries (*P* < 0.05; *n* = 5 ovaries per treatment) as determined by one-way ANOVA followed by Dunnett’s multiple comparisons test

There is some evidence that steroid hormones can cause delays in meiotic progression. We wanted to know if E_2_ or P_4_ could delay arrival at the diplotene stage in mouse ovaries. To test this, 17.5 dpc fetal ovaries were placed in organ culture for four days with control media or media containing 10^−6^ M E_2_, 10^−6^ M P_4_ or both 10^−6^ M E_2_ and 10^−6^ M P_4_. These hormone doses were chosen based on our previous studies as doses that reduced primordial follicle assembly in organ culture [14]. After culture, serial sections were prepared, stained with hematoxylin and eosin and analyzed. There was no difference in the number of oocytes between control and treated ovaries (Fig. 4a). There was also no difference in the percent of single oocytes, a measure of primordial follicle formation (Fig. 4b). In addition, there was also no statistically significant difference in the percentage of oocytes at the diplotene stage of prophase I (Fig. 4c). However, when only oocytes already assembled in primordial follicles were examined, there were significantly fewer follicles containing diplotene oocytes in the P_4_ treated ovaries (Fig. 4d).

## Discussion

Here we investigated the relationship between two critical events in mammalian oogenesis, primordial follicle formation and diplotene arrest. We confirmed that the earliest diplotene oocytes were observed at 17.5 dpc and gradually increased over time. In previous studies from our lab, the earliest age that primordial follicles were reported was 17.5 dpc [6]. However, here we observed a small percentage of oocytes in follicles even earlier, at 16.5 dpc. We also found primordial follicles containing pre-diplotene oocytes, supporting the idea that oocytes do not need to reach diplotene arrest before they can become enclosed in primordial follicles. Furthermore, oocytes within individual germ cell cysts do not appear to proceed through meiosis synchronously. Finally, progesterone treatment delayed meiotic progression of oocytes in follicles.

Borum in 1961 reported that mouse oocytes begin to reach the diplotene stage at 17.5 dpc in the Street inbred strain of mice [2]. A more recent study using CD1 outbred mice reported that the first diplotene oocytes were observed at 18.5 dpc [18]. However, for that study, the sample size was limited to only 200 oocyte nuclei at each age. We reexamined this question in the same strain (CD1) and found oocytes in diplotene arrest as early as 17.5 dpc in agreement with the original studies of Borum. Further, we see an increase in the percent of diplotene oocytes from PND1 to PND3 and no further increase to PND5. Previous reports suggest that most oocytes have arrested in the diplotene stage by PND5 and consistent with this in our studies 97% of the oocytes were in diplotene at PND5.

Inhibition of the synaptonemal complex protein SYCP1 in rats led to premature arrival of oocytes at the diplotene stage and also resulted in accelerated primordial follicle assembly [11]. These observations suggested that diplotene arrest is developmentally linked with follicle formation in rodents. Supporting this notion, Wang and colleagues found that in newborn mice, all oocytes in primordial follicles were at the dictyate (late diplotene) stage and no follicles containing earlier stages were observed [19]. However, our results here suggest that meiotic stage and primordial follicle formation are not closely linked. *Stra8* deficient female mice have been previously shown to have a meiotic initiation block at 13.5 dpc to 14.5 dpc [20]. The authors postulated that if oocytes were required to enter meiosis for follicles to assemble then *Stra8* deficient ovarian germ cells would not undergo follicle formation or activation. However, the germ cells in *Stra8* deficient ovaries did become enclosed in granulosa cells and developed to advanced stages [21]. Thus, some studies suggest that progression of oocytes to the diplotene stage is a prerequisite to form follicles in rodents while other studies including data presented here do not support this idea. In cattle, up to 80% of oocytes in primordial follicles are at prediplotene stages during fetal development suggesting that oocytes are not required to reach the diplotene stage before follicles are able to form in bovine ovaries [16]. Further investigation is necessary determine the exact link between primordial follicle formation and meiotic stage.

Here, we examined the effects of E_2_ and P_4_ on progression of oocytes to the diplotene stage. Surprisingly, P_4_ but not E_2_ delayed meiotic progression, decreasing the percent of follicles containing diplotene oocytes. In previous studies, exposure of pregnant C57BL/6 females to the estrogenic compound BPA, disrupted the process of meiotic prophase [15]. Oocytes from female fetuses had defects in synapsis and increased recombination resulting in higher levels of aneuploidy. This difference may be due to differences in the specific time period examined or route of hormone exposure. BPA exposure spanned 11.5 dpc to 18.5 dpc, while the fetal ovaries in our study were harvested at 17.5 dpc and exposed to hormones in organ culture for 4 days. There may also be differences depending on which estrogenic compound is used. Finally, Susiarjo and colleagues examined chromosomal defects while we examined progression to the diplotene stage.

Previous work from our lab demonstrated that exposure to P_4_ or E_2_, as well as synthetic estrogens, BPA, diethylstilbestrol or ethinyl estradiol delayed germ cell cyst breakdown and primordial follicle formation [14, 22]. In addition, estrogens at some concentrations also altered the oocyte loss that accompanies cyst breakdown. Here we found that P_4_ or E_2_ had no effect on cyst breakdown, primordial follicle formation or oocyte survival. The previous studies began hormone exposure at PND1 while in the current work hormone treatment began earlier at 17.5 dpc and this may be outside the window of sensitivity. Interestingly, in a previous study using rats, P_4_ but not E_2_ significantly inhibited primordial follicle assembly [23]. In our study neither hormone affected follicle assembly, however, we observed an effect of P_4_ on meiotic progression. It is also notable that there was no significant effect of P_4_ and E_2_ together on meiotic progression. We would expect that since P_4_ alone affects meiosis, both hormones would also have an effect. E_2_ may activate signals that block the effects of P_4_.

Here, we examined nuclear morphology using classic histology to analyze meiotic prophase I of perinatal oocytes so that we could address meiotic progression in the context of follicle formation and development. We wanted to use molecular tools such as antibodies that recognize meiotic marker proteins. While there are several such antibodies, most are used in the surface spread technique where oocyte nuclei are spread out and tissue structure is lost so that stage of follicle development for oocytes cannot be determined. Unfortunately, many of the antibodies do not work well in whole mount immunocytochemistry where follicle formation and development could be analyzed. We are in the process of testing meiotic prophase marker antibodies to identify one that does work in whole mount immunocytochemistry to be used in future studies.

## Conclusions

In summary, the processes of primordial follicle formation and meiotic progression to the diplotene stage do not appear to be closely linked in mouse ovaries. P_4_ reduced the percent of oocytes within primordial follicles that have reached diplotene arrest. In women, diplotene arrested oocytes often remain dormant for many years before being activated. Mechanisms controlling progression of oocytes through prophase I and arrest at the diplotene stage are not well understood but this knowledge will be essential to prevent defects in meiosis such as aneuploidy.
